# Mechanical Adaptations of Epithelial Cells on Various Protruded Convex Geometries

**DOI:** 10.3390/cells9061434

**Published:** 2020-06-09

**Authors:** Sun-Min Yu, Bo Li, Steve Granick, Yoon-Kyoung Cho

**Affiliations:** 1 Center for Soft and Living Matter, Institute for Basic Science (IBS), Ulsan 44919, Korea; estheryu0502@unist.ac.kr (S.-M.Y.); libotc@gmail.com (B.L.); sgranick@gmail.com (S.G.); 2 Department of Biomedical Engineering, Ulsan National Institute of Science and Technology (UNIST), Ulsan 44919, Korea; 3 Department of Chemistry, Ulsan National Institute of Science and Technology (UNIST), Ulsan 44919, Korea

**Keywords:** 3D geometry, epithelial cell, morphology, actin contractility, vimentin, keratin, tubulin, mechanobiology

## Abstract

The shape of epithelial tissue supports physiological functions of organs such as intestinal villi and corneal epithelium. Despite the mounting evidence showing the importance of geometry in tissue microenvironments, the current understanding on how it affects biophysical behaviors of cells is still elusive. Here, we cultured cells on various protruded convex structure such as triangle, square, and circle shape fabricated using two-photon laser lithography and quantitatively analyzed individual cells. Morphological data indicates that epithelial cells can sense the sharpness of the corner by showing the characteristic cell alignments, which was caused by actin contractility. Cell area was mainly influenced by surface convexity, and Rho-activation increased cell area on circle shape. Moreover, we found that intermediate filaments, vimentin, and cytokeratin 8/18, play important roles in growth and adaptation of epithelial cells by enhancing expression level on convex structure depending on the shape. In addition, microtubule building blocks, α-tubulin, was also responded on geometric structure, which indicates that intermediate filaments and microtubule can cooperatively secure mechanical stability of epithelial cells on convex surface. Altogether, the current study will expand our understanding of mechanical adaptations of cells on out-of-plane geometry.

## 1. Introduction

The shape of epithelia affects the physiological function [1,2,3] and supports homeostasis [4] of various epithelial organ system. Loss of original shape in epithelia is frequently related with diseases; for instance, the largely expanded cysts in renal tubule is a sign of polycystic kidney disease [1], which induces the malfunction of kidneys. Besides, a recent finding suggested that the tubular diameter of epithelial organ can determine patterns of tumorigenicity [5], demonstrating tumorigenesis can be affected by geometric features of epithelial lumens. Although the mounting evidence emphasize the importance of shape in native tissue, our understanding on the shape effect in epithelial system is still elusive.

It has been revealed that planar confined geometry can determine the growth [6], differentiation [6,7,8,9,10,11], and tumorigenicity [12] of cells by controlling their two-dimensional (2D) interfacial tension [6,12]. While 2D geometry effects have been widely studied using various micropatterning technologies, the 3D geometry effects on cells have been less explored [13]. For instance, 3D native tissue exhibits complex features such as curved tissues, winding tubules, sphere structure, etc. [14], in which the cell behaviors are influenced by various factor such as size, shape, curvature, and mechanical properties of tissue [15,16,17].

Several reports found that curved surface dictates shape change [18,19,20,21], growth [22], migration [23,24,25], and differentiation [2] of epithelial tissue. However, previous in-vitro studies were mainly conducted on cylindrical shape of the structure, which is a highly simplified design compared to shape complexity inside a real organ. For instance, renal tubules exhibit a convoluted shape of tubule, where this anatomical structure supports the functions of the kidney [26]. Recently, it has been reported that curved tubule induced the detachments of epithelial tissue cultured in alginate hollow tubule, and this propensity was more likely occurred when cells experienced high contractility and local curvature of the winding tubule [27]. However, it is still largely unexplored how the cells respond on out-of-plane geometry, even though it is essential as a cell-instructive material parameter. For instance, previous studies revealed 3D villus architecture controls intestinal stem cell localization by physically distorting the shape of the morphogenic field, causing local maxima of epithelial signals, at the tip of each villus [2]. Moreover, the unique shape of epithelial cells during tissue bending, called ‘scutoid’, was found that makes possible the minimization of the energy and stabilize three-dimensional packing [21].

Biophysical studies of cells on 3D structure remains challenging partly owing to the difficulty of quantitative tuning of the 3D geometry and measuring of cellular features on 3D structure. In the current study, we prepared the precisely fabricated 3D structure using two-photon polymerization (TPP)-laser lithography and quantitatively analyze the features of cells. Here, we asked question how epithelial cells adapt to convex geometry and which cellular factors is critically involved for this process. We studied the morphological adaptation of epithelial cells cultured on different protruded geometry to see the corner effect. In addition, we found that actin stress fibers and intermediate filaments are involved in the shape change and growth of epithelial cells. Especially, this study reveals that the intermediate filament (vimentin and cytokeratin 8/18) and microtubules(α-tubulin) are important cellular components supporting the growth and adaptation of epithelial tissue on 3D structure.

## 2. Materials and Methods

### 2.1. Fabrication of Protruded Convex Structure

**Figure 1:**
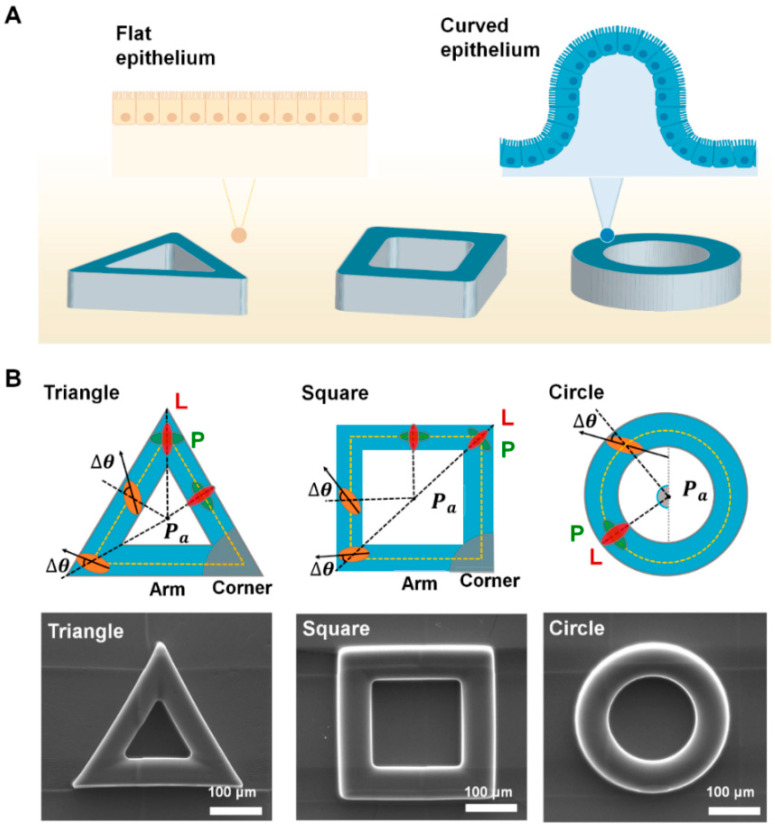
The shape of epithelial tissue and fabricated convex geometric structure. (**A**) A schematic of flat and curved epithelium on the designed protruded geometry; triangle, square and circle shape, respectively. The height and diameter of triangle, square, and circle ring structure is fixed as 60 μm. For the cell culture, fibronectin was fully covered on both protruded structure and flat surface. (**B**) The schematics showing cell orientation (∆*θ*) on the structure. ∆*θ* were measured from main axis of cells from the center of each structure (*P_a_*); triangle, square, circle geometry. When the cells are oriented from reference axis from *P_a_* in longitudinal (L) or perpendicular (P) direction, ∆*θ* equals 0° or 90°, respectively. The scanning electron microscope (SEM) images of the fabricated structure are shown in the bottom panel.

We generated smooth polydimethylsiloxane (PDMS) structures via soft lithography from a master mold fabricated by two-photon polymerization (TPP)-laser lithography (Figure 1B). The mold, having different shapes of 3D indentations (triangle, square, circle shape), was designed using SolidWorks 2019 software (Dassault Systèmes, Vélizy-Villacoublay, France). Other than geometry of the structure, we fixed other parameters (wall thickness: 60 µm; height: 60 µm). The 3D structure was fabricated using a direct laser writing system (Photonic Professional, Nanoscribe GmbH, Karlsruhe, Germany) with IP-S resin from the laser assisted nano engineering (LANE) lab at University of Nebraska–Lincoln (http://lane.unl.edu/). Then, the structure was exposed to vaporized trimethoxy (octadecyl)silane (Sigma-Aldrich, St. Louis, MO, USA) for 2 h to repetitively produce 3D convex PDMS structures (10:1 ratio of Sylgard 184 silicone elastomer base to curing agent; Dow Corning, Midland County, MI, USA). After the degassing step, we incubated it at 65 °C in a dry oven for 6 h and gently peeled off the final PDMS structures. For cell culture, the molded PDMS structure was sterilized with 70% ethanol washing, and subsequently exposure under ultraviolet lights for 30 min.

### 2.2. Surface Characterization of 3D Structure

For surface characterization of final PDMS structure, we performed scanning electron microscopy (SNE4500M; SEC Co., Ltd., Suwon, Korea) after the bare PDMS samples were coated with gold using a sputtering system (MCM-100; SEC Co., Ltd., Suwon, Korea). To measure the surface roughness of the final PDMS structure, atomic force microscopy (Dimension Icon; Bruker, Billerica, MA, USA) was conducted (Supplementary Figure S1). The AFM probe (SCM-PIT-V2; Bruker, Billerica, MA, USA) had a spring constant of 3.0 Nm^−1^. Data analysis was performed using Gwyddion AFM analysis software (Czech Metrology Institute, Brno, Czech Republic).

### 2.3. Cell Culture and Growth

We cultured wild-type Madin–Darby canine kidney cells (MDCK-WT; MDCK NBL2; ATCC, Manassas, VA, USA) in low-glucose Dulbecco’s Modified Eagle’s Medium containing 10% fetal bovine serum and 1% penicillin-streptomycin. For the maintenance, the medium was changed every 3 days, and the cells were sub-cultured at nearly 90% confluence using 0.25% trypsin (Gibco, Grand Island, NY, USA). For cell culture on the 3D geometric surface, all sterilized PDMS molds were covered with 10 μg/mL^−1^ fibronectin solution for 1 h at 25 °C for surface coating. Then, the structures were washed with phosphate buffered saline (PBS) to remove residual fibronectins in the solution. Finally, harvested MDCK cells were dispensed into a 6-well culture plate at a seeding density of 10^4^ cm^−2^ and then cultured for 3 days until full confluency.

### 2.4. Cell Immunofluorescence

We prepared cell samples for fluorescence imaging as follows. Cells were fixed with 3.7% paraformaldehyde for 15 min following gentle PBS washing. Fixed cells were permeabilized with 1% Triton-X in PBS and washed twice with PBS. Blocking was performed with 5% bovine serum albumin (BSA) in PBS solution for 30–40 min. Phalloidin-fluorescein isothiocyanate (FITC; 1:500 dilution: Sigma-Aldrich, St. Louis, MO, USA) was applied for 40 min to stain F-actin. For vimentin intermediate filaments, vimentin-Alexa648 (1:1000 dilution; Abcam, CAM, UK) was incubated in 1% BSA for 2 h at room temperature. Cell nuclei were stained with 4′,6-diamidino-2-phenylindole (DAPI; Sigma-Aldrich, St. Louis, MO, USA). For cytokeratin 8/18, mouse monoclonal anti-cytokeratin 8/18 (C51) primary antibody (1:100 dilution; Cell Signaling Technology, MA, USA) labeling was performed in 1% BSA in PBS for 2 h at room temperature. Secondary antibody labeling with goat anti-mouse IgG antibody-Alexa594 (1:1000 dilution; Abcam, CAM, UK) was performed in 1% BSA in PBS for 1 h at room temperature. For α-tubulin, mouse monoclonal anti-α-tubulin antibody (1:1000 dilution; Sigma-Aldrich, St. Louis, MO, USA) labeling was performed in 1% BSA in PBS for 2 h at room temperature. Secondary antibody labeling with goat anti-mouse IgG antibody-Alexa594 (1:1000 dilution; Abcam, CAM, UK) was performed in 1% BSA in PBS for 1 h at room temperature.

### 2.5. Confocal Fluorescence Imaging

Confocal fluorescence imaging was performed using either Carl Zeiss LSM 700 confocal laser scanning microscope with 20× objective lens (Plan-Apochromat 20×/0.8 NA; Oberkochen, Germany) or Olympus FV3000 confocal laser scanning microscope (Oylmpus, Shinjuku, Tokyo, Japan). Microscope operation and imaging was conducted using ZEN software (Zeiss, Wetzlar, Germany) or FLUOVIEW system (Olympus, Shinjuku, Tokyo, Japan). Freshly prepared fluorescent cell samples were flipped down to the cover glass, and fluorescent images were acquired at 2-µm intervals from the top surface of the structure to the bottom flat surface.

### 2.6. Imaging Analysis

The digitized individual cell information was analyzed by home-built interactive data language (IDL) (ITT visual information solutions, Boulder, CO, USA) code in a semi-automatic manner. The detail information on process and principle of analysis method were separately written in supplementary information (see Supplementary Note). To confirm that enhanced biomarker expression on 3D geometry does not originated from image artifacts from the structure protrusion, we analyzed the fluorescence signals from the BSA-FITC-coated PDMS block at distances of 0 and 60 µm from the cover slide glass using ImageJ software (National Institutes of Health, Bethesda, MD, USA) (Supplementary Figure S2). We randomly defined a rectangular region of interest of 250 µm^2^ and then averaged the FITC intensity value of the substrate at the remote distance (60 µm) compared to that of the direct contact (0 µm) condition (Supplementary Figure S2). In addition, we also measured BSA-FITC fluorescence intensity profile on circle-shape structure having wall thickness and protrusion height of 60 µm each. The fluorescence signal of each image was analyzed from the center of circle toward the radial direction using ImageJ. The averaged fluorescence intensity along the structure indicates that there is no significant signal enhancement by the protruded structure on the structure (Supplementary Figure S3A,B).

### 2.7. Cell Morphological Index and Mathematical Correction

We computed digitized cell information from 2D projected image and applied mathematical correction to feature out original morphological features of cells on convex surface. For the triangle and square structure, we further divide the surface into corner and arm (Figure 1B). To retrieve the actual morphological features of cells, we applied the following equations with first-order approximation. The relative angle (Δ*θ*) of cells was calculated as follows

(1) $$\Delta \theta =\left|\theta_{a}-\;\theta_{b}\right|$$

where $\theta_{a}$ is the orientation between the cell mass center and center of structure ($P_{a}$) (Figure 1B). $\theta_{b}$ is the orientation of the cell body, represented by the orientation of the main axis from the ellipsoidal fitting. Thus, if the cell aligned in the longitudinal or perpendicular direction from reference axis, ∆θ reached 0° or 90°, respectively.

Morphological information can be obtained from 2D projected images and angle γ, which denotes the angle formed by the line joining the cell center mass and the center of the curved surface with respect to the radius of the curved surface (see Supplementary Note). Thus, the original data of the curved surface were approximated from raw data of $\theta \prime_{a}$ and $\theta \prime_{b}$ with the following equations:

(2) $$\theta_{a}=\tan^{-1}\left(\frac{\tan\theta_{a}\prime }{\cos\gamma }\right),\;\;\theta_{b}=\tan^{-1}\left(\frac{\tan\theta_{b}\prime }{\cos\gamma }\right)$$

Since, $∆\theta =\left|\theta_{a}-\;\theta_{b}\right|$

(3) $$∆\theta =\tan^{-1}\left(\frac{\tan\theta_{a}-\tan\theta_{b}}{1+\tan\theta_{a}\tan\theta_{b}}\right)$$

The actual cell area (S) was computed over the plane of projection, as reported by Latorre et al. [28]:

(4) $$S=A_{p}\overset{N_{pix}}{\underset{i}{\sum }}\frac{1}{\cos\gamma_{i}}$$

where $N_{pix}$ represents the number of pixels in the projected area of a cell, $A_{p}$ represents the actual area unit of one pixel, and $\gamma_{i}$ denotes the angle between the line joining the center of the *i*-th pixel and the center of the curved surface (see Supplementary Note).

### 2.8. Pharmacological Interventions

To perturb actin contractility, cells were treated with 120 nM concentration of actin polymerization inhibitor Latrunculin A (Lat-A) (L5163; Merck, Denver, CO, USA) and 1 µg/mL Rho activator II CN03 (CN03-A; Cytoskeleton, Denver, CO, USA) during the culture periods. We also treated the cells with 2 mM of acrylamide (A9099; Merck, Denver, CO, USA) to disrupt the vimentin intermediate filaments organization. Afterward, cell samples were prepared for fluorescence imaging and the confocal fluorescence imaging was conducted to observe the cell behaviors by the mentioned inhibitors.

### 2.9. 3-(4,5-Dimethylthiazol-2-yl)−2,5-Diphenyltetrazolium Bromide Assay

To confirm that Lat-A cytotoxicity effects on MDCK cell growth, we checked cell viability with 3-(4,5-Dimethylthiazol-2-yl)−2,5-Diphenyltetrazolium Bromide (MTT) assay by using Lat-A concentration of 120 nM and 240 nM. (Supplementary Figure S4). The cells, seeded in a 96-well plate and cultured for 48 h with of 120 nM and 240 nM Lat-A, were assayed for cell viability according to the manufacturer’s instructions of CyQUANT^TM^ MTT Cell Viability Assay (V13154; Invitrogen, Carlsbad, CA, USA). Fresh medium without phenol-red was added 1 h before the addition of 10 μL of 12-mM MTT dye. After incubation for 4 h in MTT dye, 100 μL of stop solution was added to solubilize the formazan product. Following overnight incubation at 37 °C in 5% CO_2_, the absorbance was analyzed using a microplate reader (Tecan Infinite^®^ 200 PRO; Männedorf, Switzerland).

### 2.10. Data Analysis

The presented data are expressed as the mean ± standard error of the mean. The number of experiments and total number of cells analyzed were displayed on the figures. Each experiment was repeated at least three times. The Chen-Shapiro normality test was performed to determine the normal distribution of sample data. The Levene’s test was used to assess the equality of variances for a variable calculated for two or more groups. The statistical analysis was performed using one-way ANOVA test with post-hoc Tukey’s honest significant difference (HSD) or Fisher’s least significant difference (LSD) and two-tailed Student’s *t*-test; *p*-values < 0.05 were considered significant. Statistical significance was indicated as follows; **** *p* < 0.0001; *** *p* < 0.001; ** *p* < 0.01; * *p* < 0.05; NS, not significant. All statistical tests and graphs were performed using Excel (Microsoft, Redmond, WA, USA) and OriginPro (OriginLab, Wellesley, MA, USA).

## 3. Results

### 3.1. Epithelial Cells Exhibit the Adaptive Morphology on Convex Geometric Structure

**Figure 2:**
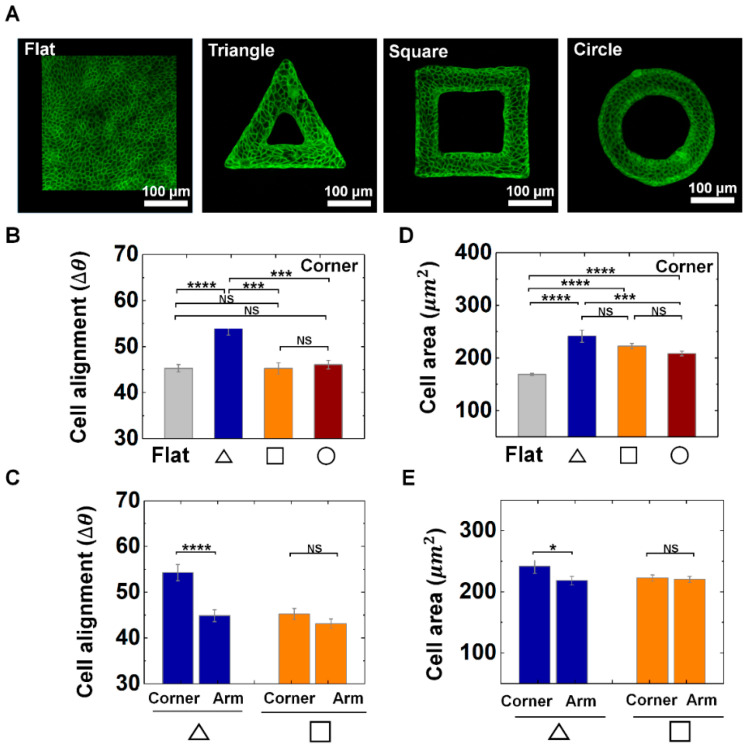
Adaptive morphology of epithelial cells on convex geometric structure. (**A**) Representative fluorescence images of MDCK cell morphology on flat and protruded structures. F-actin (green) was stained with phalloidin-FITC. (**B**) Averaged cell alignments (∆*θ*) of the cells on corner of triangle structure (navy), square (orange), and circle (red) shape. (**C**) Averaged ∆*θ* of MDCK cells on corner and arm of triangle (navy) and square (orange) structure. (**D**) Averaged area of the cells on corner of triangle structure, square and circle shape. (**E**) Averaged area of MDCK cells on corner and arm of triangle and square structure. Number of analyzed cells ($N_{cell}$) for flat surface and corner of triangle, square, circle structure was 1014 and 212, 471, 749, respectively (*N* = 3). $N_{cell}$ for arm of triangle and square structure were 412 and 580 (*N* = 3). Error bars in graph indicate the standard error of mean (S.E.M.). One-way ANOVA test with post-hoc Fisher’s least significant difference (LSD) was used. **** *p* < 0.0001; *** *p* < 0.001; * *p* < 0.05; NS, not significant.

Epithelial cells, which are building blocks of epithelial tissues, constantly coordinate their shape to induce large tissue rearrangements that eventually caused transition from planar epithelial sheets to curved forms during organ development. The anatomical shape of epithelium structurally supports the physiological functions of organs (Figure 1A). To investigate the corner effect of protruded convex geometry, we designed triangle, square, and circle shapes with a wall thickness of 60 μm (Figure 1A,B). We generated the smooth elastomeric convex scaffold (Supplementary Figure S1) generated from TPP indentation mold. For the cell culture, fibronectin solution was fully covered on both 3D structure and planar surface. We used MDCK cells, which is a prototype of epithelial cells. For 3 days, MDCK cells were cultured on outer surface of the structure to be reached confluence. Alignments and area of individual cells was analyzed to portray cellular morphological features (Figure 1B and Figure 2).

We found that cell alignment responds to the corner most significantly when they are on the triangle structure. This is indicated by the quantity Δθ. As shown in Figure 2A–C, the average Δθ of the cells indicates more perpendicular orientation near the corner of triangle structure than that of square, circle structure or flat surface, indicating the local alignments of epithelial cells on sharp corner in triangle. For instance, the average Δθ of cells on arm of the triangle structure showed 45°, while that on the triangular vertex showed 54° (Figure 2C), while we could not find significant difference in ∆θ of corner and arm part of square shape (Figure 2A–C). It demonstrates that alignments of cells in confluent epithelial tissue can respond to local features of the convex geometry. In addition, cells on the convex structures showed a greater area than that of the flat surface (Figure 2D), and cells on the corner of the triangle shape exhibited larger cell area than that of square and circle shape, indicating epithelial cells can increase the area in sharp corner regions. Moreover, the cell area in triangle shape showed differences between the corner and arm region, while the cells in square shapes did not present significant difference between corner and arm part (Figure 2E). In addition, we could observe center-oriented alignments and a gradual increase of the cell area on the circle structure as the wall thickness decreased (Supplementary Figure S5). This result indicates that both the shape and size of the protruded 3D structure influence the cell to cell interaction and their adaptive morphology of the epithelial cells. Altogether, we observed that the epithelial cells showed characteristic morphology at corner of triangle shape by showing a greater change in the alignment and area than that of the square and circle shape, demonstrating local geometric cues can tune the epithelial cell morphology on convex structure.

### 3.2. Actin Contractility Controls the Morphological Adaption of Epithelial Cells on Protruded Convex Structure

**Figure 3:**
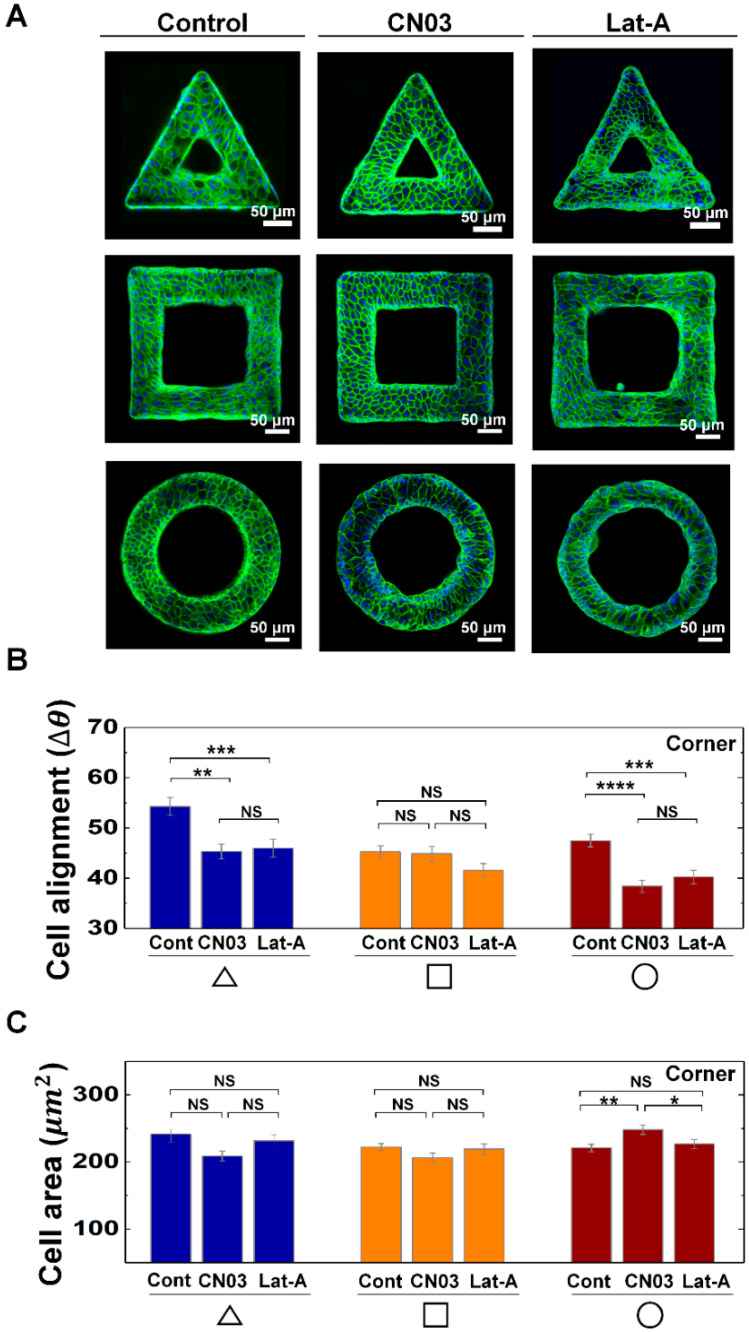
Actin contractility controls the morphological adaptation of MDCK cells on convex geometric structure. (**A**) Representative fluorescence images of MDCK cells on the 3D geometry treated with CN03 and Lat-A. F-actin (green) and nucleus (blue) were stained with phalloidin-FITC and 4′,6-diamidino-2-phenylindole (DAPI), respectively. Averaged (**B**) ∆*θ*, and (**C**) area of the cells on corner of triangle (navy), square (orange), and circle (red) structure with CN03 and Lat-A. $N_{cell}$ for CN03 treatment on the triangle, square and circle structure were 198, 364, and 430, respectively (*N* = 3). $N_{cell}$ for Lat-A treatment on the triangle, square and circle structure were 287, 343, and 435, respectively (*N* = 3). Error bars in graph indicate S.E.M. One-way ANOVA test with post-hoc Fisher’s LSD was used. **** *p* < 0.0001; *** *p* < 0.001; ** *p* < 0.01; * *p* < 0.05; NS, not significant.

We then explored the biological origins that caused the morphological adaption. To probe the relationship between cell contractility and cell morphological changes on the geometric structure, we controlled actin contractility of MDCK cells with CN03 and Lat-A (Figure 3A). The cell alignments on the corner of the triangle and circle shapes were affected by CN03 and Lat-A, while the cells on the square shape do not show significant changes. For example, the perpendicular alignment of cells on the corner of the triangle shape disappeared by either increasing or decreasing cell contractility, when it is compared with control, while the cells on circle shape showed longitudinal alignments with CN03 and Lat-A treatments (Figure 3B), demonstrating cell alignments can be affected by both cell contractility and geometry. However, there was no significant area changes on triangle and square structures with drug treatments (Figure 3C). On the circle structure, enhanced actin contractility induced larger cell area than that of control or Lat-A treated condition (Figure 3C), indicating actin contractility play important roles in maintaining cell area on circle shape structure. In addition to that, cells on the arm of triangle and square structure did not show specific changes in cell alignments and area with CN03 and Lat-A treatments, indicating the cell morphology changes on the arm of the structures were insensitive to cell contractility controls (Supplementary Figure S6). By increasing CN03 concentration, we observed that cells on circle shape structure gradually increased cell area and preferentially oriented main axis toward center of circle shape. However, no significant changes were observed in both alignment and area of the cells on triangle or square structures upon the increase of the concentration of Rho activator II CN03 from 1.0 µg/mL to 2.0 µg/mL (Supplementary Figure S7).

### 3.3. Vimentin and Keratin Intermediate Filaments are Essential for Growth and Adaptation of Epithelial Cells on Convex Geometric Structure

Vimentin intermediate filaments (IF) play important roles in the mechanical adaptation of cells by controlling cell stiffness and elasticity. Considering the multiple roles of vimentin IFs under diverse physical stimuli, we asked a question whether vimentin IF play a critical role on growth and adaptation of epithelial cells on convex surface. We found that vimentin IFs play important roles in growth and adaptation of epithelial cells. Importantly, the epithelial cells increased vimentin expression to respond local geometry of the structure.

**Figure 4:**
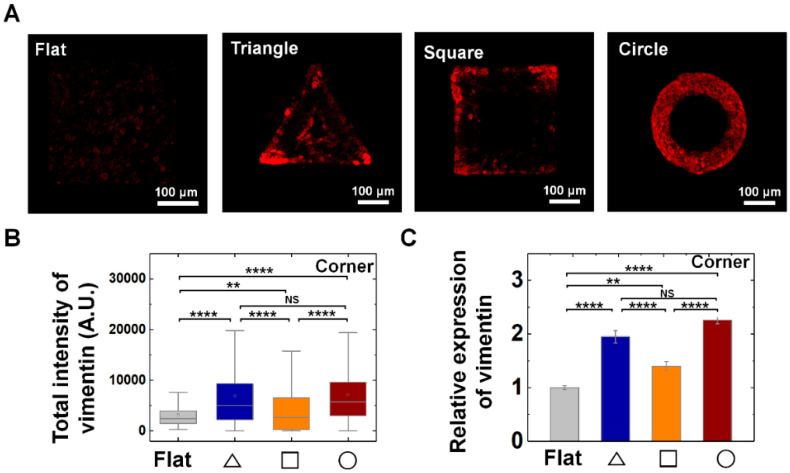
Enhanced vimentin expression on convex geometric structure. (**A**) Representative fluorescence images of vimentin (red) in MDCK cells on the flat and triangle, square, circle structure. (**B**) The box plots indicate the total intensity of vimentin per individual cells on corner of triangle (navy), square (orange) and circle (red) structure. The result from flat surface is plotted in gray color as a control (*N* = 3). (**C**) Mean relative vimentin intensity of the cells on the geometric structure compared to that on the flat surface. $N_{cell}$ for flat surface and triangle, square, circle structure was 457 and 306, 473, 919, respectively (*N* = 3). Error bars in graph indicate S.E.M. One-way ANOVA test with post-hoc Fisher’s LSD was used. **** *p* < 0.0001; ** *p* < 0.01; NS, not significant.

The common enhancements of vimentin expression of the individual cells on all three structures strongly suggests the general role it plays for the establishment and maintenance of the epithelium structure on convex geometry (Figure 4). Interestingly, we observed that vimentin was significantly enhanced at the corner region of triangle and square shape than that of arm parts (Figure 4A,B and Supplementary Figure S8A,B). For instance, the relative expression level of vimentin showed 2.0-, 1.4-, and 2.3-fold higher level at triangle, square, and circle structure than that of the flat surface (Figure 4C); while the arm part showed similar level with that of flat surface (Supplementary Figure S8A,B), indicating the cells can respond to local geometry of 3D structure by showing distinctive pattern of vimentin expression. Especially, unlike local vimentin expression at corner of triangle and square shape, the cells on the circle shape showed homogeneous expression of vimentin following the circular structure, suggesting curvature continuity is an important factor in mechanical adaptation of cells with vimentin IFs (Figure 4).

**Figure 5:**
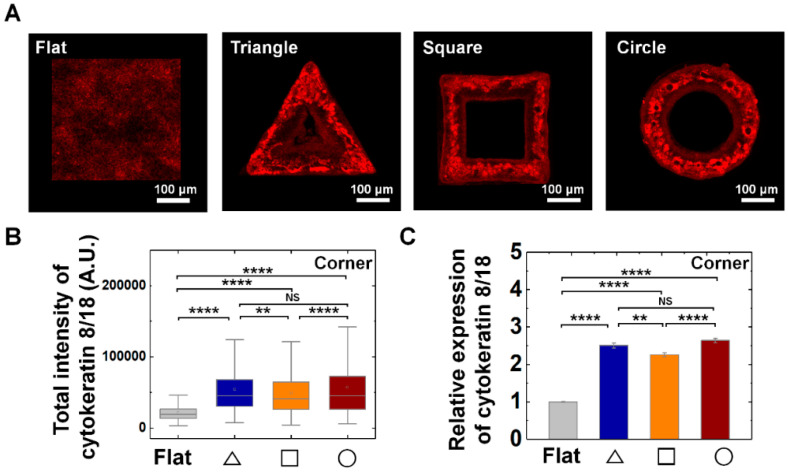
Enhanced cytokeratin 8/18 expression on convex geometric structure. (**A**) Representative fluorescence images of cytokeratin 8/18 (CK8/18; red) in MDCK cells on the flat and triangle, square, circle structure. (**B**) The box plots indicate the total intensity of CK8/18 per individual cells on corner of triangle (navy), square (orange), and circle (red) structure. The result from flat surface is plotted in gray color as a control (*N* = 5). (**C**) Mean relative CK8/18 intensity of the cells on the geometric structure compared to that on the flat surface. $N_{cell}$ for flat surface and triangle, square, circle structure was 4761 and 571, 799, 1324, respectively (*N* = 5). Error bars in graph indicate S.E.M. One-way ANOVA with post-hoc Fisher’s LSD was used. **** *p* < 0.0001; ** *p* < 0.01; NS, non-significant.

In addition, we further checked the epithelial specific cytokeratin (CK) IFs, CK 8/18 expression on all three-structure types. We confirmed CK8/18 expression of MDCK cells were enhanced on convex geometric structure; the relative expression level of CK8/18 showed 2.5-, 2.3-, and 2.6-fold higher level at triangle, square, and circle structure than that of flat surface (Figure 5). Interestingly, unlike the pattern of vimentin IFs expression, CK 8/18 exhibited homogeneous expression both on the corner and arm part of the structure (Supplementary Figure S8C,D).

**Figure 6:**
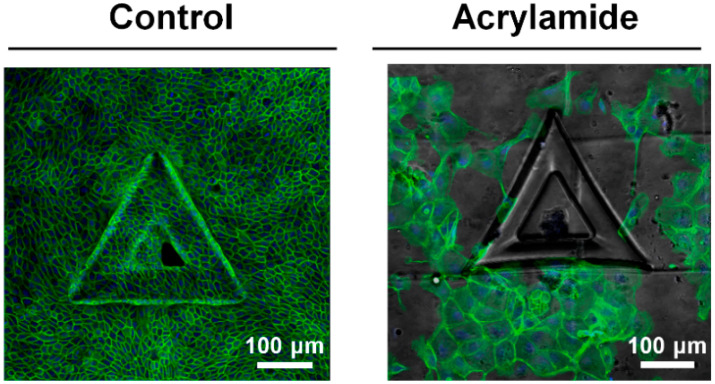
Disturbing vimentin intermediate filaments (IFs) networks influence cell growth and adaptation on convex structure. Representative fluorescence images of MDCK cells after 3 days of incubation without- and with 2mM acrylamide, causing collapse of vimentin filaments organization. F-actin (green) and nucleus (blue) were stained with phalloidin-FITC and DAPI, respectively.

The effect of IFs in epithelial cell growth was further confirmed with inhibitor experiments. The treatments of acrylamide, disturbing IFs organization, impeded the growth of MDCK cells on convex surface, which showed less confluent, disorganized cell growth results (Figure 6). While the control samples completely covered the structure and adjusted on the structure within three days, the acrylamide treatment impeded the growth of cells on the structure (Figure 6). Altogether, we could see IFs are important cellular components which enable growth of the cells on convex structures by sustaining the cellular integrity. Altogether, this result indicates the epithelial cells are sensitive to local geometric features of 3D structure such as edge and curvatures of the structure and control the vimentin expression to adapt accordingly.

### 3.4. Tubulin, Microtubule Buildng Blocks, Involves in Mechanical Adaptation of Epithelial Cells on Convex Geometric Structure

**Figure 7:**
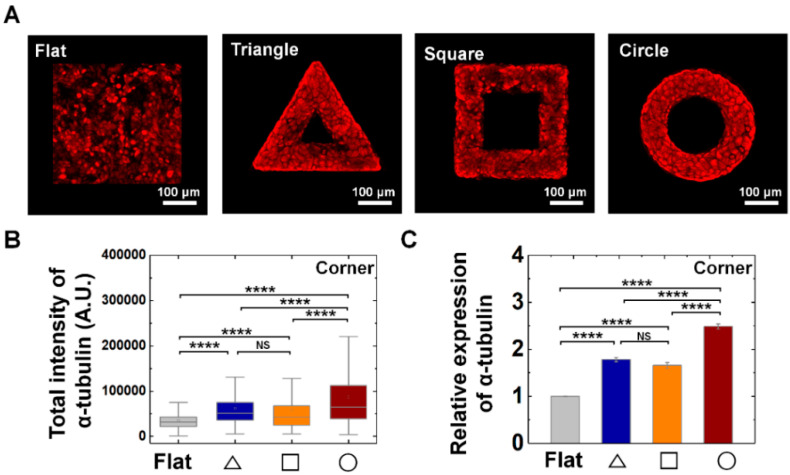
Enhanced α-tubulin expression on convex geometric structure. (**A**) Representative fluorescence images of α-tubulin (red) in MDCK cells on the flat, triangle, square, and circle structure. (**B**) The box plots indicate the total intensity of α-tubulin per individual cells on corner of triangle (navy), square (orange), and circle (red) structure. The result from flat surface is plotted in gray color as a control (*N* = 6). (**C**) Mean relative α-tubulin intensity of the cells on the geometric structure compared to that on the flat surface. $N_{cell}$ for flat surface and triangle, square, circle structure was 4893 and 603, 704, 1487, respectively (*N* = 6). Error bars in graph indicate S.E.M. One-way ANOVA with post-hoc Fisher’s LSD was used. **** *p* < 0.0001; NS, non-significant.

From the vimentin and keratin expression, we found that two types of intermediate filaments responded to convex geometric structure. Since it is well-known about the major group of cytoskeletons as actin, microtubule, intermediate filament, we further studied whether mechanical adaptation of epithelial cells also can be related with microtubule organization. We confirmed α-tubuln expression, microtubule building unit, on convex geometric structure. We confirmed that α-tubulin expression was enhanced on the structure than that of flat surface. For instance, the relative expression level of tubulin showed 1.8-, 1.7-, and 2.5-fold higher level at triangle, square, and circle structure than that of flat surface, suggesting microtubule also involves in mechanical adaptation of epithelial cells on out-of-plane structure (Figure 7). Especially, another characteristic of α-tubulin expression is homogeneous expression on both corner (Figure 7) and arm (Supplementary Figure S8E–F) parts of the structure.

## 4. Discussion

The structure of epithelial organs is tightly controlled by the morphological and mechanical features like cell polarity [29], cell–cell adhesion intersection [30], and morphogen gradients [31]. The loss of shape in epithelial organs is closely correlated with cancer and several diseases [32]. In this study, we found that epithelial cells respond to local geometry by showing characteristic morphological changes on convex geometric structure. We revealed the adaptation of epithelial cells on protruded convex structure by controlling geometric features: triangle, square, and circle shape. We found that the epithelial cells showed characteristic morphology at corner of triangle shape, when it is compared with a flat surface, by showing greater changes in alignment and area than that of square and circle shape, which demonstrates local geometric cue can tune the epithelial cell morphology on convex structure. However, we observed that overall cell area was decreased regardless of the type of the geometry and all reached the similar value of the flat surface when we extended the culture time from 3 to 4 days (Supplementary Figure S9). This result indicates that higher cell density by longer cultivation time neutralize the morphological changes of cells induced by the protruded structure of the substrate, which suggests the cell to cell interaction along the lateral direction also plays an important role in the epithelia on various shapes of out-of-plane structure.

The rho-activator, CN03, constitutively activates Rho by blocking GTPase activity and does not interfere with Rac or Cdc42 activity [33,34], thereby regulating the stress fiber formation and actomyosin contractility [35]. To inhibit the actin polymerization, we treated Lat-A, which depolymerizes the actin stress fibers and attenuates cell contractility [36]. From the inhibitor experiments, we observed that characteristic morphological adaptation of epithelial cells on 3D geometry can be controlled by actin contractility, especially cell alignments on triangle and circle shape showed significant difference when the cell contractility was perturbed by CN03 or Lat-A. However, there was no significant area changes on triangle and square structure with these drug treatments; while enhanced actin contractility induced larger cell area on circle geometry, indicating actin contractility play important roles in maintaining cell area on circle shape structure.

In additions, we found that the depolymerizing IFs impeded the epithelial cell growth on convex surface, demonstrating the essential role of IFs in epithelial cell adaptation on out-of-plane structure. Vimentin IF is responsible for cell shape control [37], integrity [38] of the cytoplasm, and stable cytoskeletal interactions [39]. Importantly, the vimentin expression at corner of geometric structure indicates the epithelial cells sense the local geometry of the convex surface. Besides, circle shape induced relatively enhanced and homogeneous vimentin expression than that of the triangle one, suggesting importance of curvature continuity in curvature adaptation of epithelial cells. Previous studies reported the hyperelastic vimentin IFs support mechanical stability of living cells, thus protecting cells against mechanical damage [38]. In addition, the results of keratin IFs expression indicate that not only vimentin and CK8/18 also participate to mechanical response of epithelial cells to convex geometry. It has been reported that mechanical stability and integrity of epithelial cells and tissues were supported by keratin IFs [40]. Recently, it has been reported that force-induced recruitments of cten (tension 4) along keratin IFs, not actin stress fibers, in epithelial cells, indicating existence of a mechanotransduction pathway via keratin network [41]. Considering biophysical roles of vimentin and keratin, our study shows that IFs plays important roles in epithelial tissue growth on convex surface, the expression of which can be related with adaption strategy of epithelial cells by securing the mechanical stability. Concerning the tubulin expression, we found that variety of cytoskeletons can be closely involved in epithelial cell adaptation on convex surface in a cooperative manner. Previous findings reported that interactive modulation of microtubule and actin to intermediate filaments organization, suggesting microtubule is also involved in mechanical adaptation of epithelial cells on out-of-plane structure [40]. Particular interesting point of cytoskeleton marker expression is distinctive patterns of each marker (actin, tubulin, vimentin, keratin) on the structure, which is completely different from non-biological patterns of protein coating, which suggest distinctive roles of each cytoskeleton proteins on 3D shape (Supplementary Figure S3C). Taken all together, this result demonstrates that the mechanoresponse of epithelial cells on 3D structure is an important cue in shaping tissue architecture and supporting epithelial cell adaptation on an out-of-plane structure [31,42].

It has been widely known that 2D geometry can affect diverse cellular responses such as cell shape, proliferation, differentiation, mechanoresponsive marker expression, and underlying mechanism has been suggested that confined adhesive area which can be tuned by geometry of 2D patterns cause interfacial tension at the boundary [6,12]. As both topography and adhesiveness of the surface may play essential roles in cellular behavior [43], we treat the whole surface of the 3D geometry to be adhesive so that we can elucidate the roles of 3D geometry independent of chemical adhesiveness.

Several studies can be further explored regarding on 3D geometry effect on cell behaviors. Actomyosin is tightly coupled with cell contractility generation [15], myosin interaction can elucidate further how actomyosin contractility controls the epithelial cells response on 3D geometric structure. Biophysical roles of vimentin IFs present possible correlation with activation of mechanotransduction marker, such as Yes-associated protein (YAP) in growth and adaptation of epithelial cell on 3D space.

Altogether, our results demonstrate that epithelial cells can sense the sharpness of corners of the 3D structure by showing the characteristic response in cell alignments and area change. By controlling actin polymerization reaction, we found that cell alignments at the corner of the structure were sensitive to actin contractility, while the cell area only increased on circle shapes when cell contractility was enhanced. More importantly, we found that not only actin filaments, but vimentin and keratin intermediate filaments, play important roles in cell growth and adaptation of epithelial cells on convex structure. Besides, tubulin expression indicates cooperative modulation of cytoskeleton during the growth of epithelial tissue on out-of-plane geometry. Especially, enhanced vimentin expression at the corner region indicates epithelial cells can sense and respond to local geometric elements. These results increase our understanding of mechanical adaptations of cells on convex structure, reflecting in vivo 3D tissue architecture in diverse biological processes such as organogenesis, tubulogenesis, and tumor metastasis.

## Supplementary Materials

The following are available online at https://www.mdpi.com/2073-4409/9/6/1434/s1, Supplementary Note: Image analysis process. Figure S1: The roughness characterization of fabricated polydimethylsiloxane (PDMS) structure, Figure S2: BSA-FITC fluorescence intensity measurement of flat PDMS surface with defined distance, Figure S3: Fluorescence intensity profile of BSA-FITC and cytoskeleton expression on circle-shape structure, Figure S4: Quantification of cell viability using MTT assay upon Latrunculin A (Lat-A) treatments, Figure S5: Morphology of MDCK cells on circle shape convex structure as a function of wall thickness, Figure S6: Morphology of MDCK cells on arm of triangle and square shape structure with CN03 and Lat-A treatments, Figure S7: Morphology of MDCK cells on the corner of triangle, square, and circle shape structure with CN03 treatment, Figure S8: The expression of vimentin, keratin intermediate filaments and α-tubulin on arm of triangle and square structure, Figure S9: Area of epithelial cells on convex geometric structure with a various shape at 4 days of cell cultivation.
