# Supplementary material for: Mechanical Adaptations of Epithelial Cells on Various Protruded Convex Geometries
Source: Cells. 2020 Jun 9;9(6):1434. doi: 10.3390/cells9061434 (PMC7349491; doi:10.3390/cells9061434)
Supplement: Supplementary file 1 [file cells-09-01434-s001.pdf]

Supplementary Information for

Mechanical Adaptations of Epithelial Cells on Various Protruded Convex Geometries

Sun-Min Yu [1,2,†], Bo Li [1,†], Steve Granick [1,3] and Yoon-Kyoung Cho [1,2,*]

[1] Center for Soft and Living Matter, Institute for Basic Science (IBS), Ulsan 44919, Korea; estheryu0502@unist.ac.kr (S.-M.Y.); libotc@gmail.com (B.L.); sgranick@gmail.com (S.G.)

[2] Department of Biomedical Engineering, Ulsan National Institute of Science and Technology (UNIST), Ulsan 44919, Korea

[3] Department of Chemistry, Ulsan National Institute of Science and Technology (UNIST), Ulsan 44919, Korea

* Correspondence: ykcho@unist.ac.kr; Tel.: +82-52-217-5524

† These authors contributed equally to this work

Received: 10 April 2020; Accepted: 5 June 2020; Published: date

This PDF file includes:

Supplementary Note

Image analysis process

Supplementary Figures

Figures S1 to S9

Supplementary Note

Image analysis process

In the main text, we reported how the protruded structure influences the morphology and biomarker expression of the cells. Here we show how this information is quantitatively extracted from the raw image of the confocal microscopy using the imaging analysis codes.

As reported in the Methods section, 3D confocal fluorescence images of the cells on protruded structures were obtained and then the 3D confocal images were reconstructed in IMARIS software (Bitplane, Zürich, Switzerland). From that, we generated 2D projected-single plane image (**Figure SN1A**) by applying 2D projection function where the reconstructed 3D objects can be orthogonally projected in XY plane direction with maximum intensity projection (MIP). This 2D projected image (**Figure SN1A**) is then the starting point of our home-built IDL codes that digitalize the morphology and biomarker expression of every individual cells.

The first step is to segment the cells that are in confluent status using the watershed algorithm. As shown in **Figure SN1A**, the F-actin (Phalloidin-FITC, green signal) localizes well at the boundary of the cells. This feature allows us segmenting the whole image (**Figure SN1A**) into individual patches (**Figure SN1B**) and obtaining the boundary of each patch (**Figure SN1C**). Note that each channel (green, blue and red) of a colored image is a 1000-pixel by 1000-pixel matrix and the magnitude of the matrix element is the intensity of the corresponding signal (e.g., green for F-actin and blue for nucleus). Therefore, after the watershed segmentation, we obtain the pixels' coordinate that consist of both the boundary and the region enclosed by the boundary of every cell. Two representative cells (the two cells in the colored boxes (red and purple) in **Figure SN1C**) extracted from the raw image are schematically shown in **Figure SN1D**. The solid curves (red and purple) are the cell boundary. The green and blue area that fill the areas inside the solid curves represent the green and blue signals in the raw image (**Figure SN1A**), respectively. In the following steps, we discuss how to quantify the biomarker expression and the cell morphology using the boundary (red and purple curves) and inner cell information (green and blue patches) as schematically presented in **Figure SN1D**.

To obtain the level of biomarker expression, we cropped the part of the raw image (**Figure SN1E, F**) that corresponds to the pixels that are identified to belong to the inner cell region (green areas in **Figure SN 1D**). For a certain type of marker, we further select the pixels whose brightness magnitude in this channel (e.g, green for F-actin, blue for nucleus) is greater than an empirical threshold value. The expression level of this biomarker is then the average value of these further selected pixels. The reason for setting a threshold value is to get rid of the white noises of the equipment like CCD.

For the morphology quantities, the calculation procedures are presented as below. The perimeter and area of the cells are obtained by simply counting the number of pixels that are identified as cell boundary (red and purple curves in **Figure SN1D**) and inner cell region (green areas in **Figure SN1D**), respectively. The mass center (red and purple crosses in **Figure SN1D**) of the cells are determined by taking average of the coordinate of the pixels that are enclosed by the cell boundary, i.e., the inner cell pixel. By fitting the inner cell points into an ellipsis (dotted black curves in **Figure SN1D**), we use the orientation of the long axis (red and purple dashed lines in **Figure SN1D**) of the fitted ellipsis as the orientation of the cell body. The

relative angle is then the angle between the cell body orientation (red and purple dashed lines in **Figure SN1D**) and the orientation between the mass center of cell and the structure (origin lines in **Figure SN1D**).

Note that the cells are growing on the curved protruded structure instead of the flat surface. Therefore, the extracted morphology information needs to be corrected based the geometrical relationship between the area occupied by the cell and its xy-projection (**Figure SN1G**). The formulism of this step has already been reported in the main text (2. Materials and Method/2.6. Cell morphological index and mathematical correction) and one can refer to that section for details.

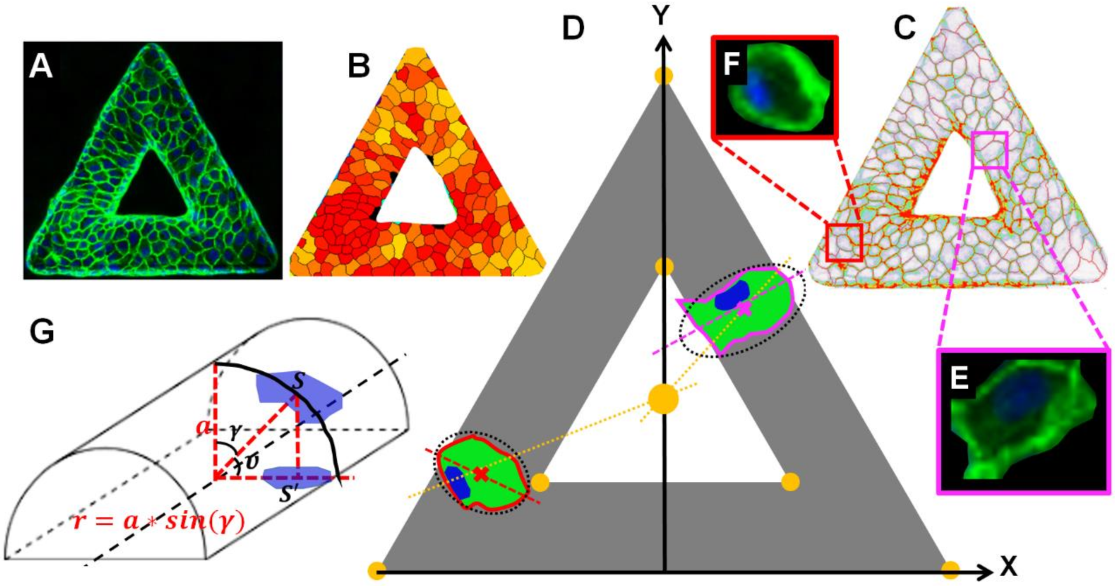

Figure SN1. Important steps of imaging analysis that extract the morphology and biomarker expression of cells. (A) 2D projected-single plane image of MDCK cells on the triangular structure. **(B)** The mosaic patches that represent the regions that occupied by every cell. **(C)** The boundary of the cells obtained from the watershed segmentation. **(D)** The schematics of two cells (corresponding to the two boxes in C) showing their morphology and orientation on the structure. The solid curves (red and purple) are the cell boundary. The green and blue area that fill the areas inside the solid curves represent the green and blue signals in the raw image of (A**),** respectively. The orange dots are the vertexes and center of the triangular structure and the orange dotted lines are the reference lines that are parallel to the corresponding arm of the triangular. The colored crosses (red and purple) are the cell mass center obtained by averaging the coordinates of all pixels that are enclosed by the boundary curves. The dotted black ellipsis are the fit results of the boundary points by ellipsoid fitting. The colored dash lines (red and purple) are the long axis of the ellipsis, which represent the orientation of the cells. **(E, F)** The crop of the raw images that corresponding to the region enclosed by the boundary points obtained from the watershed segmentation. **(G)** Schematic representations of parameters for the correction of original cell morphology on curved surface. In the scheme, v indicate the angle of point on the surface from the center of curved surface, respectively. The area obtained from the projected image is corrected to the real area by dividing the value of $\cos(v)$.

Supplementary Figures

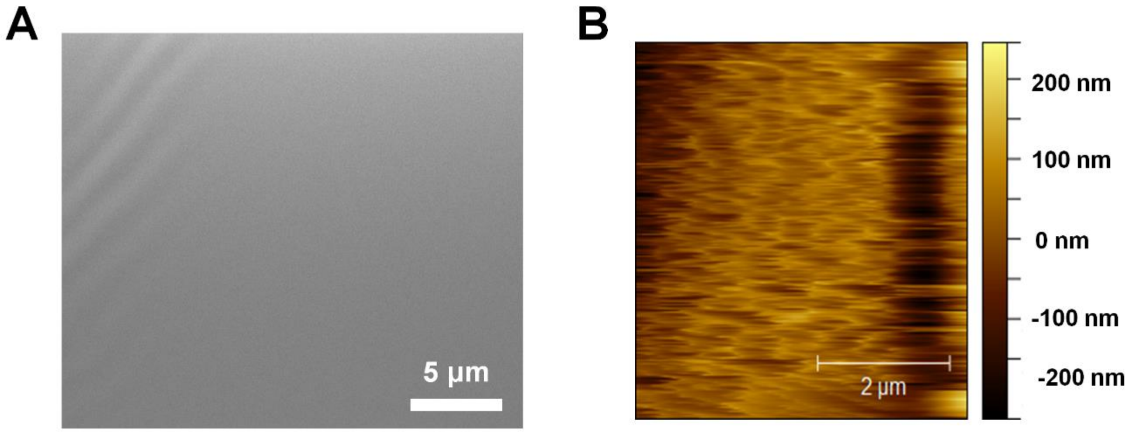

Figure S1. The roughness characterization of fabricated polydimethylsiloxane (PDMS) structure. **(A)** The enlarged SEM images of the curved tubule shows the smooth surface. **(B)** The curved surface roughness measurement by atomic force microscopy.

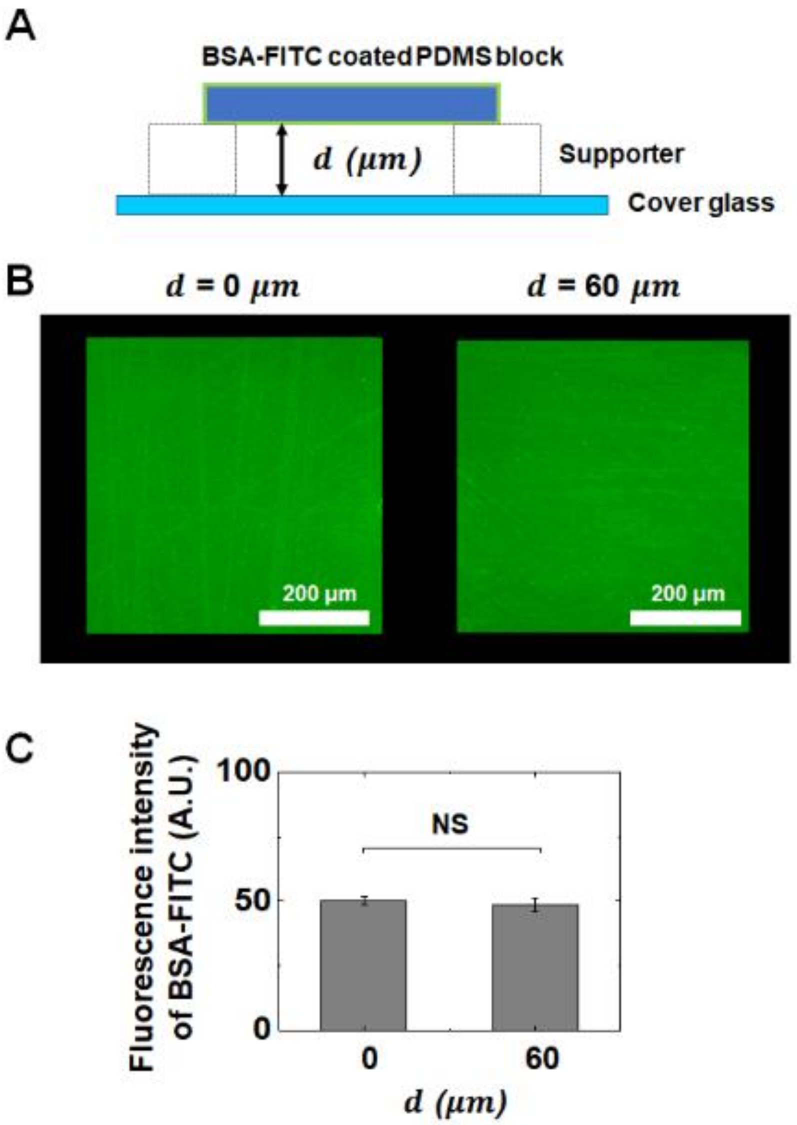

Figure S2. BSA-FITC fluorescence intensity measurement of flat PDMS surface with defined distance. (A) Graphical illustration of experiments to measure the fluorescence signal of BSA-FITC (green) on PDMS block with distance control **(B)** Representative images of PDMS flat surface coated with BSA-FITC (green) are shown at 0 and 60 μm distances from the cover glass. **(C)** Averaged fluorescence intensity level of BSA-FITC on the PDMS surface at 0 and 60 μm distances from the cover glass slide. The error bars in the graph indicate the standard error of mean (S.E.M.) (N = 5). Two-tailed Student's t-test was performed; NS, not significant

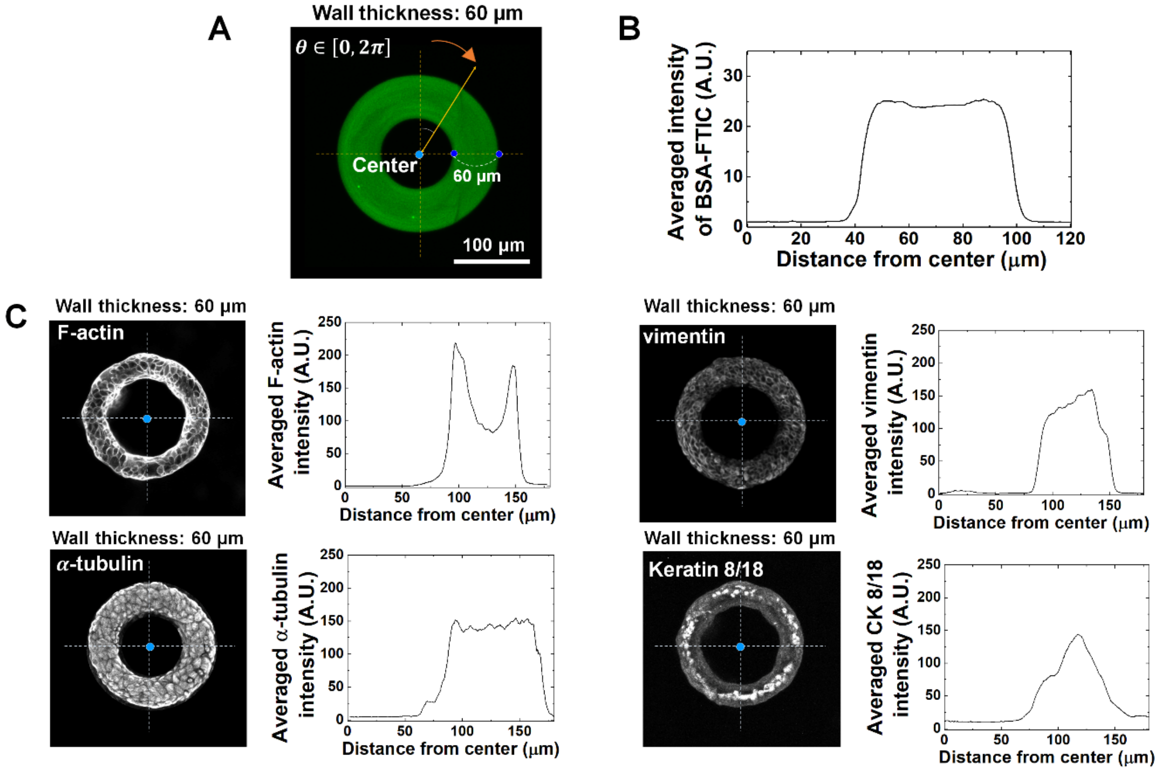

Figure S3. Fluorescence intensity profile of BSA-FITC and cytoskeleton expression on circle-shape structure. (A) Representative images of circle shape structure coated with BSA-FITC. The blue dot indicates center of the structure and wall thickness is 60 µm. **(B)** Linear plots in the middle of the figure indicate the average intensity profiles of BSA-FITC signals on the circle-shape structure as a function of distance from center (N = 3). **(C)** Representative images and fluorescence profile of cytoskeleton markers (F-actin, α-tubulin, vimentin and keratin 8/18) on circle-shape structure having wall thickness of 60 µm. The line scan profiles indicate the averaged fluorescence intensity as a function of the distance from the center (N = 3).

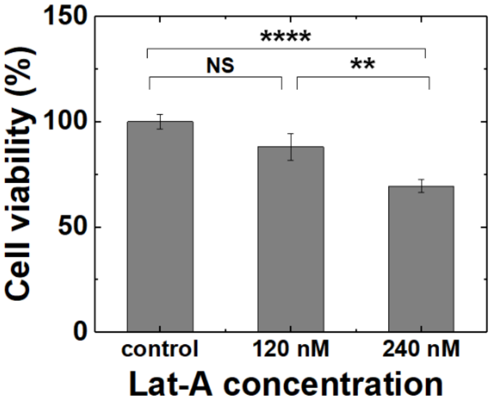

Figure S4. Quantification of cell viability using MTT assay upon Latrunculin A (Lat-A) treatments. MDCK cells were treated with 120 nM and 240 nM of Lat-A to check the cytotoxicity (N=3). Significance was determined by one-way ANOVA using Tukey's correction for multiple comparisons. ****, $p < 0.0001$; **, $p < 0.01$; NS, non-significant.

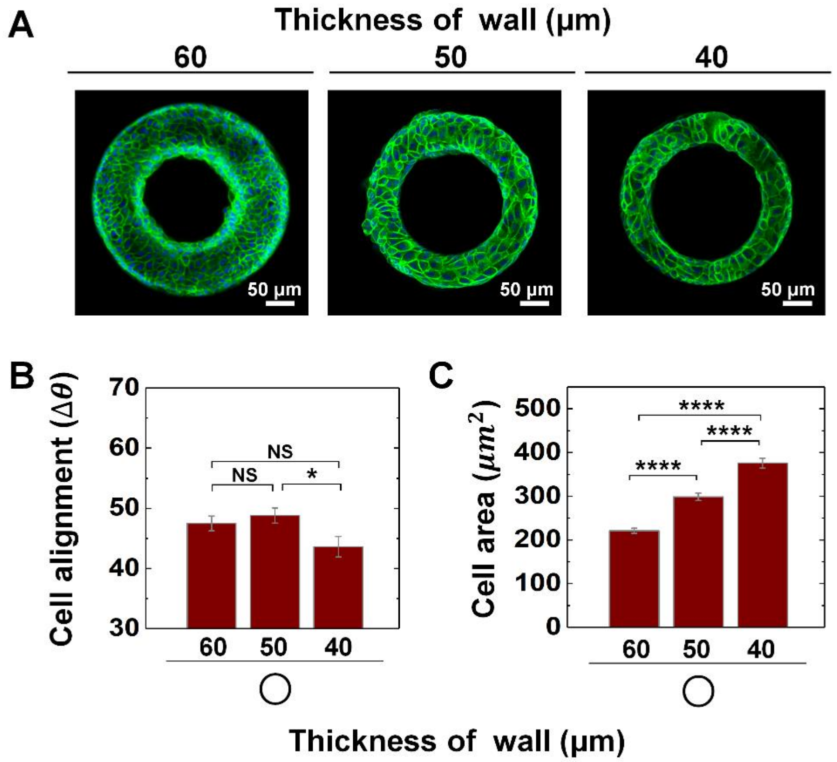

Figure S5. Morphology of MDCK cells on circle shape convex structure as a function of wall thickness. **(A)** Representative fluorescence images of MDCK cells on the 3D circle geometry having wall thickness of 60, 50, and 40 μm. F-actin (green) and nucleus (blue) were stained with phalloidin-FITC and DAPI, respectively. **(B)** $\Delta\theta$, and **(C)** area of the cells on the circle structure as a function wall thickness. N_{cell} for wall thickness of 60, 50 and 40 μm were 749, 580 and 399, respectively (N=3). Error bars in graph indicate S.E.M. One-way ANOVA with post-hoc Fisher's LSD was used. ****, $p < 0.0001$; *, $p < 0.05$; NS, not significant.

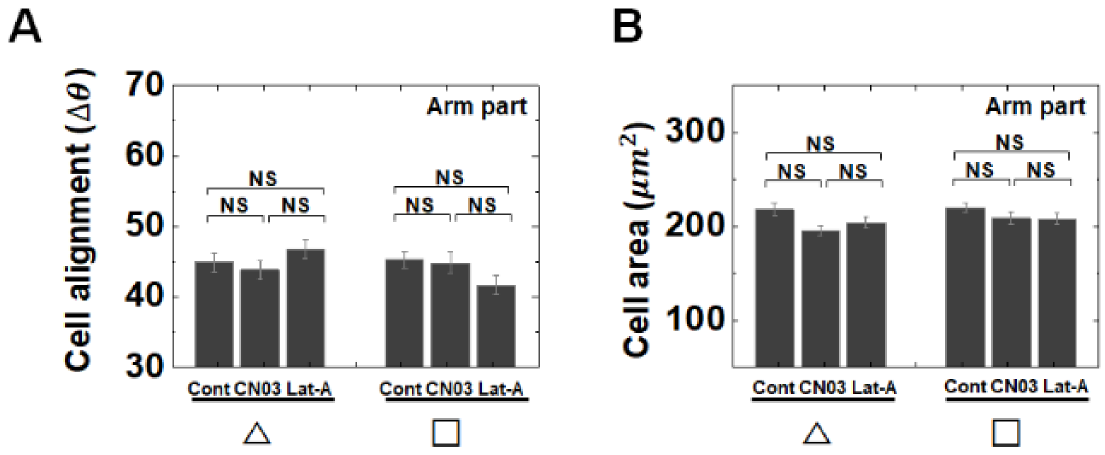

Figure S6. Morphology of MDCK cells on arm of triangle and square shape structure with CN03 and Lat-A treatments (A) Averaged cell alignments ($\Delta\theta$) and **(B)** area of MDCK cells on arm of 3D geometry treated with CN03 and Lat-A. N_{cell} for CN03 (resp. Lat-A) treatment on arm of the triangle and square structure were 340 (resp. 402) and 364 (resp. 343), respectively (*N*=3). Error bars in graph indicate S.E.M. One-way ANOVA test with post-hoc Fisher's LSD was used. NS, not significant.

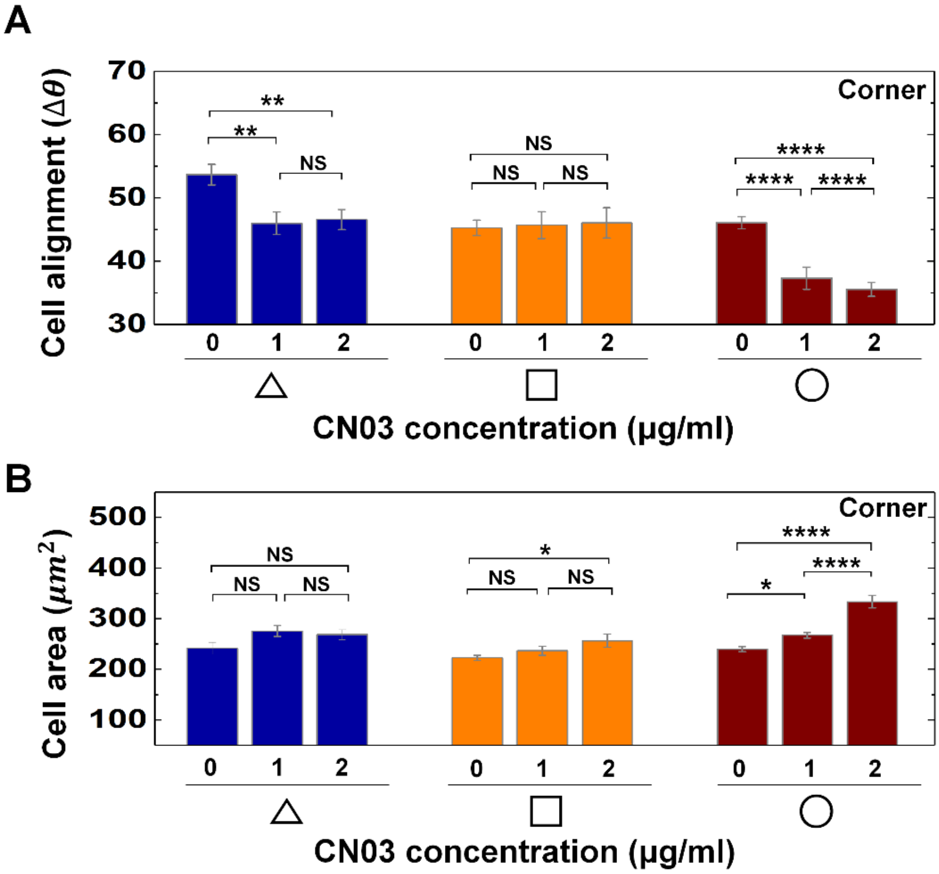

Figure S7. Morphology of MDCK cells on the corner of triangle, square, and circle shape structure with CN03 treatment (A) Averaged cell alignments ($\Delta\theta$) and **(B)** area of MDCK cells of 3D geometry treated as a function of concentration of CN03 (0, 1, 2 μg/ml). N_{cell} for control condition (no CN03 treatment) on the triangle, square and circle structure were 181, 471 and 729 respectively (N=3). N_{cell} for 1 μg/ml CN03 treatment on the triangle, square and circle structure were 254, 219 and 835 respectively (N=3). N_{cell} for 2 μg/ml CN03 treatment on the triangle, square and circle structure were 287, 139 and 570 respectively (N=3). Error bars in graph indicate S.E.M. One-way ANOVA with post-hoc Fisher's LSD was used. ****, $p < 0.0001$; **, $p < 0.01$; *, $p < 0.05$; NS, not significant.

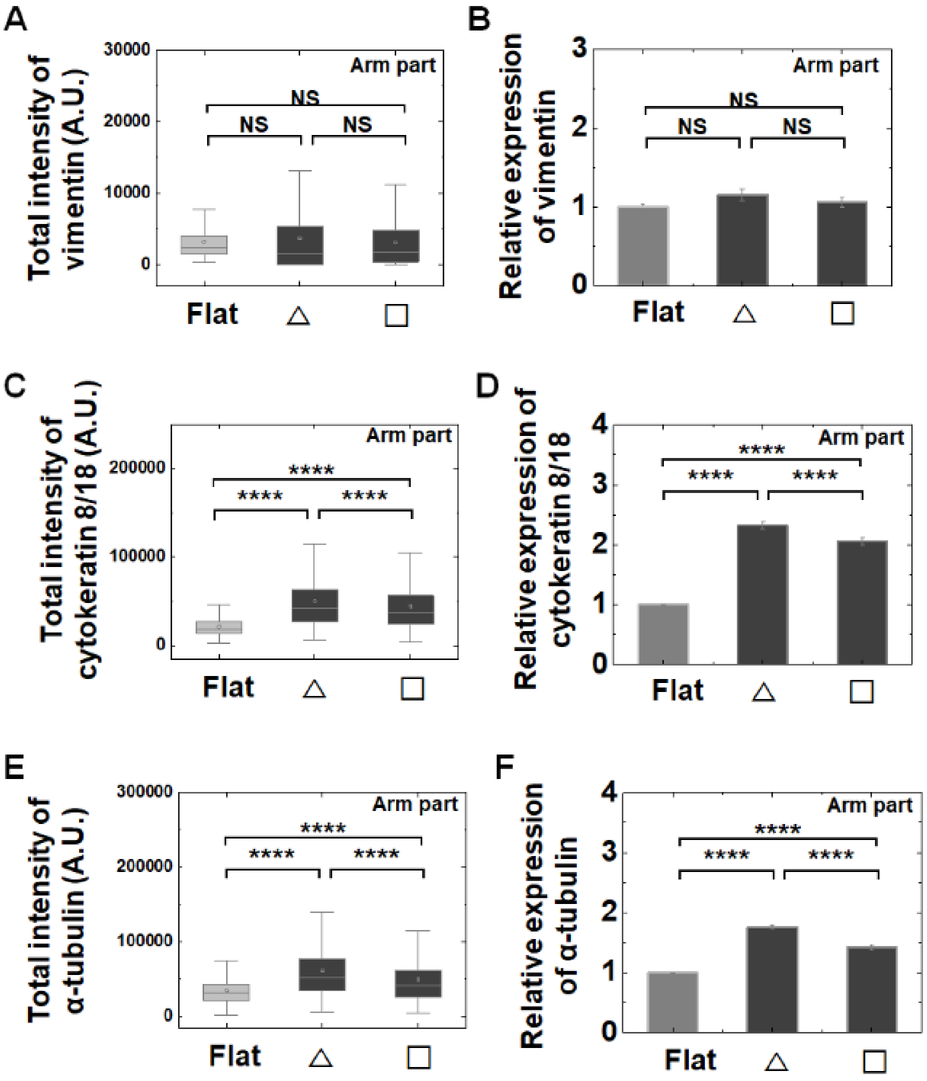

Figure S8. The expression of vimentin, keratin intermediate filaments and α-tubulin on arm of triangle and square structure (A) The box plots indicate the total intensity of vimentin per individual cells on flat surface and arm of triangle and square structure. **(B)** Mean relative vimentin intensity of the cells on the geometric structure compared to that on the flat surface. N_{cell} for flat surface and arm of triangle, square structure was 457 and 548, 428, respectively (*N*=3). **(C)** The box plots indicate the total intensity of cytokeratin 8/18 per individual cells on flat surface and arm of triangle and square structure. **(D)** Mean relative cytokeratin 8/18 intensity of the cells on the geometric structure compared to that on the flat surface. N_{cell} for flat surface and arm of triangle, square structure was 4761 and 706, 621, respectively (*N*=5). **(E)** The box plots indicate the total intensity of α-tubulin per individual cells on flat surface and arm of triangle and square structure. **(F)** Mean relative α-tubulin intensity of the cells on the geometric structure compared to that on the flat surface. N_{cell} for flat surface and arm of triangle, square structure was 4893 and 771, 634, respectively (*N*=6). Error bars in graph indicate S.E.M. One-way ANOVA test with post-hoc Fisher's LSD was used. ****, $p < 0.0001$. NS, not significant.

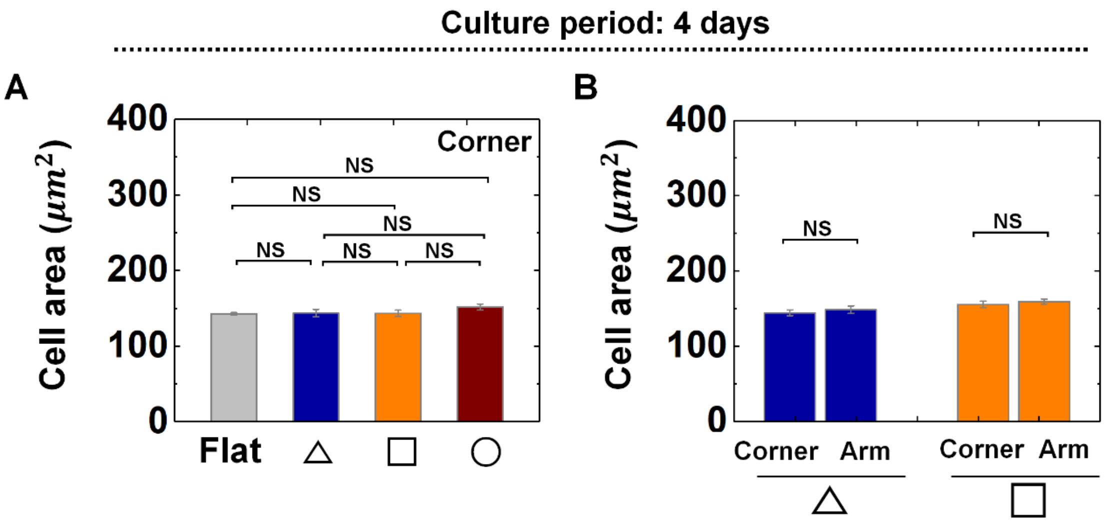

Figure S9. Area of epithelial cells on convex geometric structure with a various shape at 4 days of cell cultivation. (A) Averaged area of the cells on corner of triangle, square and circle shape structure (culture period: 4 days). N_{cell} for flat surface and corner of triangle, square, and circle structure was 1621 and 184, 389, 600, respectively (N=3). (B) Averaged area of the cells on corner and arm of the triangle and square structure (culture period: 4 days). N_{cell} for arm of triangle and square structure were 262 and 583 (N=3). Error bars in graph indicate the standard error of mean (S.E.M.). One-way ANOVA with post-hoc Fisher's LSD was used. ****, $p < 0.0001$; ***, $p < 0.001$; *, $p < 0.05$; NS, not significant.
